# Progression independent of relapse activity in relapsing multiple sclerosis: impact and relationship with secondary progression

**DOI:** 10.1007/s00415-024-12448-4

**Published:** 2024-05-28

**Authors:** Emilio Portaccio, Matteo Betti, Ermelinda De Meo, Ilaria Addazio, Luisa Pastò, Lorenzo Razzolini, Rocco Totaro, Daniele Spitaleri, Alessandra Lugaresi, Eleonora Cocco, Marco Onofrj, Franco Di Palma, Francesco Patti, Davide Maimone, Paola Valentino, Valentina Torri Clerici, Alessandra Protti, Diana Ferraro, Giacomo Lus, Giorgia Teresa Maniscalco, Vincenzo Brescia Morra, Giuseppe Salemi, Franco Granella, Ilaria Pesci, Roberto Bergamaschi, Umberto Aguglia, Marika Vianello, Marta Simone, Vito Lepore, Pietro Iaffaldano, Giancarlo Comi, Massimo Filippi, Maria Trojano, Maria Pia Amato, the Italian Multiple Sclerosis Register

**Affiliations:** 1 Department of NEUROFARBA, University of Florence, Careggi University Hospital, Florence, Italy; 2 NMR Research Unit, Queen Square Multiple Sclerosis Centre, UCL Queen Square Institute of Neurology, University College London, London, UK; 3 San Salvatore Hospital, L’Aquila, Italy; 4 AORN San G. Moscati, Avellino, Italy; 5 IRCCS Istituto Delle Scienze Neurologiche Di Bologna, Bologna, Italy; 6 Dipartimento Di Scienze Biomediche E Neuromotorie, Università Di Bologna, Bologna, Italy; 7 University of Cagliari, Cagliari, Italy; 8 University G. d’Annunzio Di Chieti-Pescara, Chieti, Italy; 9 ASST Lariana Ospedale S. Anna, Como, Italy; 10 University of Catania, Catania, Italy; 11 UOS Sclerosi Multipla, Policlinico G Rodolico-San Marco, University of Catania, Catania, Italy; 12 Centro Sclerosi Multipla, Azienda Ospedaliera Cannizzaro, Catania, Italy; 13 Institute of Neurology, University Magna Graecia, Catanzaro, Italy; 14 Fondazione IRCCS Istituto Neurologico C. Besta, Milan, Italy; 15 Niguarda Hospital, Milan, Italy; 16 Department of Neurosciences, Ospedale Civile Di Baggiovara, Azienda Ospedaliero-Universitaria Di Modena, Modena, Italy; 17 University of Campania Luigi Vanvitelli, Naples, Italy; 18 A Cardarelli Hospital, Naples, Italy; 19 Federico II University, Naples, Italy; 20 Department of Biomedicine, Neuroscience and Advanced Diagnostics (BIND), University of Palermo, Palermo, Italy; 21 University of Parma, Parma, Italy; 22 Ospedale VAIO Di Fidenza AUSL PR, Fidenza (PR), Italy; 23 IRCCS Fondazione Mondino, Pavia, Italy; 24 Department of Medical and Surgical Sciences, Magna Graecia University of Catanzaro, Catanzaro, Italy; 25 Ca’ Fancello Hospital, AULSS2 Treviso, Italy; 26 Department of Translational Biomedicine and Neurosciences, University of Bari Aldo Moro, DiBraiN, Bari, Italy; 27 Istituto Di Ricerche Farmacologiche Mario Negri IRCCS, Milan, Italy; 28 Casa Di Cura del Policlinico, Vita-Salute San Raffaele University, Milan, Italy; 29 Vita-Salute San Raffaele University, Milan, Italy; 30 IRCCS San Raffaele Scientific Institute, Milan, Italy; 31 IRCCS Don Carlo Gnocchi Foundation, Florence, Italy; 32 Pediatric MS Center, Department of Precision and Regenerative Medicine and Ionian Area, University of Bari Aldo Moro, Bari, Italy

**Keywords:** Multiple sclerosis, Relapse-associated worsening, Progression independent of relapse activity, Secondary progression

## Abstract

**Objectives:**

We investigated the occurrence and relative contribution of relapse-associated worsening (RAW) and progression independent of relapse activity (PIRA) to confirmed disability accrual (CDA) and transition to secondary progression (SP) in relapsing multiple sclerosis (MS).

**Methods:**

Relapsing-onset MS patients with follow-up > / = 5 years (16,130) were extracted from the Italian MS Registry. CDA was a 6-month confirmed increase in Expanded Disability Status Scale (EDSS) score. Sustained disability accumulation (SDA) was a CDA with no EDSS improvement in all subsequent visits. Predictors of PIRA and RAW and the association between final EDSS score and type of CDA were assessed using logistic multivariable regression and multivariable ordinal regression models, respectively.

**Results:**

Over 11.8 ± 5.4 years, 16,731 CDA events occurred in 8998 (55.8%) patients. PIRA (12,175) accounted for 72.3% of CDA. SDA occurred in 8912 (73.2%) PIRA and 2583 (56.7%) RAW (*p* < 0.001). 4453 (27.6%) patients transitioned to SPMS, 4010 (73.2%) out of 5476 patients with sustained PIRA and 443 (24.8%) out of 1790 patients with non-sustained PIRA. In the multivariable ordinal regression analysis, higher final EDSS score was associated with PIRA (estimated coefficient 0.349, 95% CI 0.120–0.577, *p* = 0.003).

**Discussion:**

In this real-world relapsing-onset MS cohort, PIRA was the main driver of disability accumulation and was associated with higher disability in the long term. Sustained PIRA was linked to transition to SP and could represent a more accurate PIRA definition and a criterion to mark the putative onset of the progressive phase.

**Supplementary Information:**

The online version contains supplementary material available at 10.1007/s00415-024-12448-4.

## Introduction

Disability accrual in patients with multiple sclerosis (pwMS) can derive from two main mechanisms, relapse-associated worsening (RAW) on the one hand, and progression independent from relapse activity (PIRA) on the other [1, 2]. RAW has been considered the paradigm of disability accrual of the relapsing, inflammatory phase of multiple sclerosis (MS), while PIRA has been considered the paradigm of disability accrual during the progressive phase of disease, either primary or secondary, sustained by neurodegenerative mechanisms [1, 2]. Recent observations have challenged the phenotypical dualism between relapsing and progressive forms of MS, showing that a ‘silent’ progression is detectable since the earliest phases of the disease, either in cohorts of patients with relapsing MS from randomized controlled trials [3, 4] or real-world cohorts [4–8]. While it is increasingly recognized that PIRA occurs in relapsing MS, it has not been fully elucidated whether RAW and PIRA can coexist in the same patient and which is the long-term impact of different combinations of RAW and PIRA on disability accumulation. Moreover, in the majority of previous studies, definitions of disability accrual events (both RAW and PIRA) were confirmed at 24–48 weeks, which reduced but did not exclude the risk of overestimation due to clinical fluctuations, different and inhomogeneous visit density and potential long-term recovery. This is particularly relevant to assess the role of RAW and PIRA events on long-term disability burden, including the transition to secondary progressive (SP) MS.

In the present multicenter study based on the Italian Multiple Sclerosis register, we, therefore, investigated the occurrence and relative contribution of RAW and PIRA to confirmed disability accrual and transition to SP, with a particular focus on sustained disability accumulations (SDA) after each event and on patients with multiple confirmed disability accrual (CDA) events.

## Materials and methods

Anonymized clinical records of patients with a first demyelinating event were extracted from the Italian Multiple Sclerosis Register [9].

The minimum dataset required for this study also comprised the main demographic characteristics, the date of disease onset, clinical course, follow-up visit dates, Expanded Disability Status Scale (EDSS) [10] scores recorded at each visit, the date of all relapses, start and end dates of all disease-modifying treatments (DMT) and DMT type. Quality assurance through online certification of EDSS competency is required at each participating site. Inclusion criteria were: CIS or RR course at the first neurological evaluation; a minimum of three visits with EDSS evaluation; a minimum of 5-year follow-up. We excluded patients with a primary progressive (PP) and SP course at the first neurological evaluation and those enrolled in randomized controlled trials. The baseline was defined as the first neurological evaluation with EDSS scoring. If the first evaluation occurred within 30 days from a relapse, baseline was defined as the following assessment with EDSS scoring performed outside of a relapse and within 1 year from the first evaluation. When re-baseline was not possible, patients were excluded. A fixed baseline EDSS was applied.

Multiple sclerosis duration was calculated from the first demyelinating event. The follow-up time was defined as the time between the first and last available EDSS evaluation. Confirmed disability accrual (CDA) was defined as ≥ 24-week confirmed disability increase from study baseline, measured by EDSS using a stepwise criterion (increase ≥ 1.5 points if baseline EDSS = 0; increase ≥ 1.0 point if baseline EDSS ≥ 1.0 and ≤ 5.5; increase ≥ 0.5 point if baseline EDSS ≥ 6.0). The date of CDA was assigned at the first EDSS score at which an increase occurred. The confirmatory EDSS score had to be above the limit of the stepwise EDSS increase as compared to baseline. In case of multiple CDA in the same patient, EDSS was re-baselined after each CDA (the EDSS at CDA became the baseline EDSS for further events). Sustained disability accumulation (SDA) was defined as a CDA with no EDSS improvement in all subsequent available visits. Any EDSS improvement after a CDA prevented the definition of SDA (no confirmation of EDSS improvement was required).

PIRA was defined as a CDA event occurring > 90 days after and > 30 days before the onset of a relapse. Otherwise, the CDA was defined as RAW. A relapse was defined as the occurrence of new symptoms or the exacerbation of existing symptoms that persisted for 24 h or more in the absence of concurrent illness or fever and that occurred 30 days or more after a previous relapse.^11^

Transition to SP was defined according to a data-driven algorithm based on a previous published definition [12] with some modifications [7]: a PIRA event with a minimum EDSS score of 4.0 at the time of conversion to SPMS and at the end of follow-up (final EDSS ≥ 4.0). For this definition, the date of PIRA event was assigned to SP conversion.

For DMT exposure, the proportion of time during which patients received DMT was defined by the recorded starting and ending dates. The total time a patient spent on treatment was calculated including any switches and gaps in treatment. We did not consider gaps < 3 months as a therapy interruption. For DMT in which extended treatment effects are recognized, the estimated treatment effect duration was used to calculate the proportion of time that patients received therapy (6 months for mitoxantrone, rituximab, ocrelizumab; 5 years for alemtuzumab and autologous haematopoietic stem-cell transplantation; 2 months for natalizumab; 12 months for cladribine) [7, 13].

### Statistical analysis

The baseline and follow-up characteristics were expressed as mean and standard deviation (SD) or frequency and percentage for continuous and categorical covariates, respectively. Categorical and continuous variables were compared using Chi2 statistic, Mann–Whitney and Kruskal–Wallis test, as appropriate.

Patients with at least 2 CDAs were grouped on the basis of the type of CDA events in patients with only RAW (oRAW), patients with only PIRA (oPIRA) and patients with RAW and PIRA (RAW + PIRA). Predictors of oRAW and oPIRA during follow-up were assessed using multivariable logistic regression models. The association between EDSS score at the last neurological evaluation and type of CDA events (oRAW versus oPIRA or RAW + PIRA) was assessed through multivariable ordinal regression model.

Results of regression analyses were expressed as odds ratio (OR) and 95% confidence interval (CI) or estimated coefficient and 95% CI, as appropriate. The multivariable modeling analyses were adjusted for the following covariates: sex (female versus male), symptom at onset (multifocal versus unifocal), age at first visit, disease duration at first visit, disease course (RR versus CIS) and EDSS score at first visit, number of relapses during follow-up, percentage of time spent on DMT during follow-up, number of EDSS evaluations during follow-up*.* The number of relapses during follow-up was included in the models to adjust for disease activity, even in patients with disability accrual independent of relapses. The multivariable ordinal regression model on EDSS score at last visit was also adjusted for the total number of CDA events.

All statistical analyses were performed with SPSS version 25.0 and R version 4.1.2. P-value < 0.05 was considered statistically significant.

## Results

**Fig. 1:**
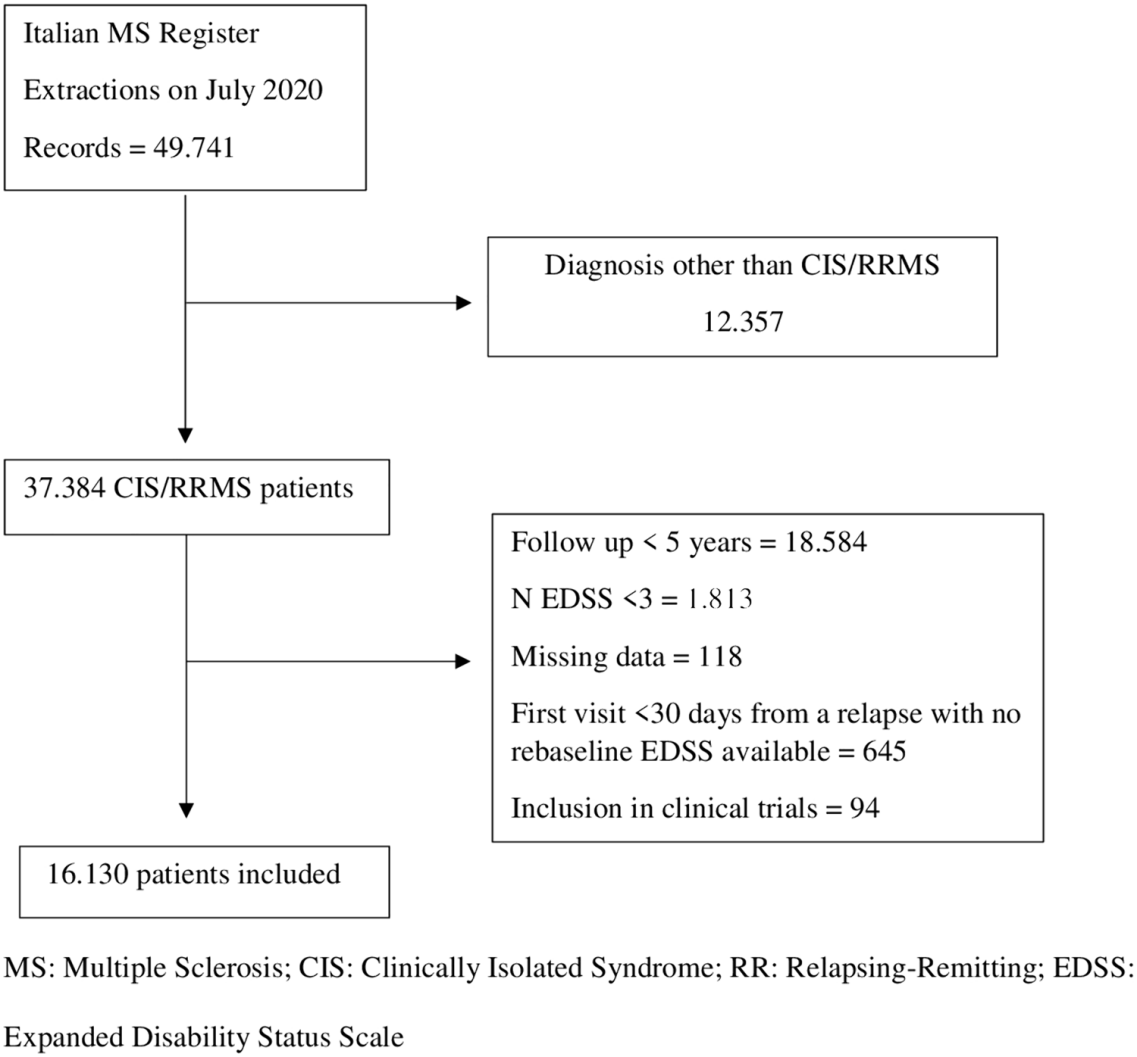
Flow chart of the study population. *MS* Multiple sclerorsis, CIS Clinically isolated syndrome, RR Relapsing remitting, EDSS Expanded disability status scale

Data extraction was completed in July 2020. We had access to 49,741 register patients from 77 Italian multiple sclerosis centers. By applying inclusion and exclusion criteria, we identified 16,130 patients (Fig. 1). Characteristics of the study sample are depicted in Table 1. Over a follow-up period of 11.8 ± 5.4 years, 16,731 CDA events occurred in 8998 patients (55.8%). Overall, PIRA (*n* = 12,175) accounted for 72.3% of CDAs and RAW for the remaining 27.7% of CDAs (*n* = 4556) (Supplementary Figure S1). PIRA events accounted for 67.2% of first CDA (2834 out of 4217), 77.0% of 2nd–4th CDAs (5507 out of 7151) and 86.9% of CDAs from 5th onwards (506 out of 582) (Supplementary Figure S1).

**Table 1:** Characteristics of the study sample

	Total sample (*n* = 16,130)
Age at baseline, year, mean ± SD	35.7 ± 10.7
Sex, female *n* (%)	11,013 (68.3)
Disease course, *n* (%)	
CIS	2687 (16.7)
RR	13,443 (83.3)
Disease duration, median (IQR)	2.8 (0.7–8.4)
EDSS, median (IQR)	2.0 (1.0–3.0)
Onset topography, *n* (%)	
Unifocal	14,068 (87.2)
Multifocal	2062 (12.8)
Follow-up duration, year, mean ± SD	11.8 ± 5.4
Number of visits during follow-up^a^, mean ± SD	22.7 ± 15.5
DMT, *n* (%)^a^	14,768 (91.6)
Percentage of follow-up^a^ spent on DMT, mean ± SD	68.8 ± 35.8
Final EDSS, median (IQR)	2.5 (1.5–5.0)

*CIS* clinically isolated syndrome, *RR* relapsing–remitting, *SD* standard deviation, *IQR* inter-quartile range, *EDSS* Expanded Disability Status Scale, *DMT* disease-modifying treatment

^a^Calculated over the entire follow-up. Starting DMT: platform DMT (interferons, glatiramer-acetate, teriflunomide, dimethyl-fumarate, and azathioprine) in 15,194 (94.2%) patients; high-efficacy DMT (cladribine, sphingosine-1-phosphate modulators, mitoxantrone, antiCD20, natalizumab, alemtuzumab) in 936 (5.8%) patients

### Impact of RAW and PIRA events and relationship of PIRA with SPMS

In the whole sample, 11,495 out of 16,731 CDA were sustained at the end of the follow-up. SDA occurred in 8912 (73.2%) PIRA and 2583(56.7%) RAW (*p* < 0.001).

Over the follow-up period, 4453 (27.6%) patients transitioned to SPMS. Among those, 3294 (73.9%) transitioned at the first, 996 (22.4%) at the second, 156 (3.5%) at the third and 7 (0.2%) at fourth PIRA event.

Focusing on 5476 patients with sustained PIRA, 4010 (73.2%) transitioned to SPMS over the follow-up. Among those, 3163 (78.9%) transitioned at the first, 548 (13.7%) at the second and 53 (1.3%) at the third sustained PIRA event. The 1466 (26.8%) patients with sustained PIRA and without transitioning to SPMS were younger (36.9 ± 10.2 vs 39.3 ± 10.8 years, *p* < 0.001), most frequently female (68.7% vs 64.4%, *p* = 0.003), with shorter disease duration (2.8 (0.7–8.7) vs 5.1 (1.6–11.4) years, *p* < 0.001), lower EDSS (1.0 (0–1.5) vs 2.5 (2.0–3.5), *p* < 0.001), with unifocal onset (87.9% vs 85.1%, *p* = 0.008), shorter follow-up duration (11.7 ± 5.1 vs 13.8 ± 6.1, *p* < 0.001) and longer exposure to DMT (69.6 ± 35.4% vs 63.1 ± 38.9%, *p* < 0.001) (Supplementary Table S1).

On the other hand, among 1790 patients with non-sustained PIRAs, 443 (24.8%) transitioned to SPMS over the follow-up. In comparison with patients with non-sustained PIRAs non converting to SPMS, they were older (38.5 ± 10.1 vs 34.9 ± 10.0 years, *p* < 0.001), with longer disease duration (5.8 (2.0–12.5) vs 2.8 (0.8–8.1) years, *p* < 0.001), higher EDSS (3.0 (2.0–4.0) vs 1.5 (1.0–2.0), *p* < 0.001), shorter follow-up duration (12.9 ± 5.1 vs 14.8 ± 6.4, *p* < 0.001) and shorter exposure to DMT (68.2% ± 36.3% vs 77.2 ± 30.2%, *p* < 0.001) (Supplementary Table S2).

### RAW and PIRA in patients with multiple CDA events

Focusing on the subgroup of patients with multiple CDA events (4217 patients), 279 (6.6%) patients had only RAW, 2100 (49.8%) had only PIRA, and 1838 (43.6%) had RAW + PIRA events. In the subgroup of RAW + PIRA patients, 1104 (60.1%) had RAW and 734 (39.9%) PIRA as first CDA. Starting from the second CDA, PIRA became predominant also in this subgroup of patients, while the proportion of RAW gradually decreased, disappearing beyond the seventh CDAs. Characteristics of patients with multiple CDAs are depicted in Table 2. Patients who developed only PIRA events were older at baseline (40 ± 10.7 years) compared to “RAW + PIRA” patients (35.4 ± 9.7 years) and to oRAW subgroup (32.8 ± 9.9 years; *p* < 0.001), had higher EDSS at baseline (2.5 (1.5–3.5) vs 2.0 (1.0–3.0) in RAW + PIRA and 2.0 (1.0–2.5) in oRAW; *p* < 0.001), longer disease duration at baseline (5.5 (1.7–11.7) years vs 3.6 (1.0–8.9) years in RAW + PIRA and 3.0 (0.7–7.5) years in oRAW, *p* < 0.001) and were less frequently treated with DMTs (91.4% versus 95.7% in RAW + PIRA versus 96.8% oRAW, *p* > 0.001). Patients in the oRAW group were more frequently female (74.6% versus 65.2% in RAW + PIRA and 65.0% in oPIRA; *p* = 0.006), had a higher number of relapses during follow-up (8 (5–13) vs 5 (3–8) in RAW + PIRA and 1 (0–3) in oPIRA; *p* < 0.001).

**Table 2:** Characteristics of patients with at least 2 CDA events (*n* = 4217)

	RAW (*n* = 279)	RAW + PIRA (*n* = 1838)	PIRA (*n* = 2100)	*p*
Age at baseline, year, mean ± SD	32.8 ± 9.9	35.4 ± 9.7	40 ± 10.7	< 0.001*
Age at onset, year, mean ± SD	27.4 ± 9	29.4 ± 9.5	32.3 ± 10.4	< 0.007*
Sex, female *n* (%)	208 (74.6)	1199 (65.2)	1366 (65.0)	0.006
Disease course, *n* (%)				
CIS	48 (17.2)	252 (13.7)	272 (13)	0.145
RR	231 (82.8)	1586 (86.3)	1829 (87)	
Disease duration, year, median (IQR)	3.0 (0.7–7.5)	3.6 (1.0–8.9)	5.5 (1.7–11.7)	< 0.001°
EDSS, median (IQR)	2.0 (1.0–2.5)	2.0 (1.0–3.0)	2.5 (1.5–3.5)	< 0.001°
Onset topography, *n* (%)				
Unifocal	238 (85.3)	1565 (85.1)	1792 (85.3)	0.986
Multifocal	41(14.7)	273 (14.9)	308 (14.7)	
Follow-up duration, year, mean ± SD	14.1 ± 6.1	15.5 ± 6.3	13.9 ± 5.8	< 0.002§
Number of visits during follow-up, mean ± SD	31.1 ± 16.4	30.5 ± 17	25.4 ± 16.7	< 0.001 ^
Number of relapses during follow-up median (IQR)	8 (5–13)	5 (3–8)	1 (0–3)	< 0.001*
DMT, *n* (%)	270 (96.8)	1759 (95.7)	1920 (91.4)	< 0.001°
Percentage of follow-up spent on DMT, mean ± SD	69.4 ± 30	63.8 ± 33	64.2 ± 36.4	0.035ç
Final EDSS, median (IQR)	5 (3.5–6.0)	6.5 (5.0–7.0)	6 (4.5–7.0)	< 0.001^

*CDA* confirmed disability accrual, *RAW* relapse-associated worsening, *PIRA* progression independent of relapse activity, *SD* standard deviation, *CIS* clinically isolated syndrome, *RR* relapsing–remitting, *IQR* inter-quartile range, *EDSS* Expanded Disability Status Scale, *DMT* disease-modifying treatment

^*^PIRA vs RAW; PIRA vs RAW + PIRA; RAW vs RAW + PIRA

^°^PIRA vs RAW; PIRA vs RAW + PIRA

^§^RAW + PIRA vs RAW; RAW + PIRA vs PIRA

^^^RAW vs PIRA; RAW vs RAW + PIRA

çRAW vs RAW + PIRA

In the multivariable logistic regression analysis (Supplementary Table S3) having only RAW events was associated with female sex (OR = 1.47; 95% CI 1.10–1.97, *p* = 0.011), younger age at baseline (OR = 0.97; 95% CI 0.96–0.99, *p* = 0.001), lower EDSS at baseline (OR = 0.87; 95% CI 0.79–0.97, *p* = 0.009), and higher number of relapses during follow-up (OR = 1.21; 95% CI 1.17–1.24, *p* < 0.001).

On the other hand, predictors of having only PIRA events (Supplementary Table S4) were older age at onset (OR = 1.01; 95% CI 1.01–1.02, *p* = 0.036), higher EDSS at onset (OR = 1.15; 95% CI 1.10–1.21, *p* < 0.001) and lower number of relapses during follow-up (OR = 0.69; 95% CI 0,67–0.71, *p* < 0.001).

EDSS at the end of follow-up was higher in oPIRA (6 (4.5–7.0)) and RAW + PIRA patients (6.5 (5.0–7.0)) compared with the oRAW subgroup (5 (3.5–6.0); *p* < 0.001).

In the multivariable ordinal regression analysis, higher EDSS score at last visit was associated with PIRA occurrence (estimated coefficient 0.349, 95% CI 0.120–0.577, *p* = 0.003), higher baseline EDSS (estimated coefficient 1.205, 95% CI 1.155–1.255, *p* < 0.001), shorter exposure to DMT during follow-up (estimated coefficient -0.841, 95% CI -1.019–0.662, *p* < 0.001), lower number of EDSS evaluations (estimated coefficient -0.029, 95% CI -0.033- -0.025, *p* < 0.001), longer follow-up duration (estimated coefficient 0.040, 95% CI 0.029–0.051, *p* < 0.001) and higher number of CDAs (estimated coefficient 1.711, 95% CI 1.641–1.782, *p* < 0.001) (Table 3).

**Table 3:** Factors associated with EDSS score at the end of the follow-up in patients with at least 2 CDAs (*n* = 4217)

	Estimated coefficient	95% CI	*p*
Only PIRA or RAW + PIRA versus only RAW	0.349	0.120–0.577	0.003
Sex (female versus male)	− 0.093	− 0.206–0.021	0.110
Onset topography (unifocal versus multifocal)	− 0.107	− 0.259–0.045	0.167
Age, years	− 0.004	− 0.011–0.002	0.157
Disease course (CIS versus RR)	− 0.071	− 0.235–0.093	0.395
Disease duration, years	− 0.004	− 0.013–0.004	0.317
EDSS	1.205	1.155–1.255	< 0.001
Percentage of time spent on DMT during follow-up	− 0.841	− 1.019 to–0.662	< 0.001
Number of relapses during follow-up	0.009	− 0.004–0.023	0.176
Number of EDSS evaluations during follow-up	− 0.029	− 0.033 to − 0.025	< 0.001
Follow-up duration	0.040	0.029–0.051	< 0.001
Total number of CDA	1.711	1.641–1.782	< 0.001

*EDSS* Expanded Disability Status Scale, *CDA* confirmed disability accrual, *PIRA* progression independent of relapse activity, *RAW* relapse-associated worsening, *CIS* clinically isolated syndrome, *RR* relapsing–remitting, *DMT* disease-modifying treatment

## Discussion

In the present multicenter, observational, retrospective cohort study based on prospectively acquired clinical data, including a large cohort of relapsing-onset MS patients followed for a mean time of 11.8 years, we assessed temporal profile and impact of RAW and PIRA and the relationship between PIRA and onset of SPMS. We also focused on patients with multiple CDA events.

As expected, PIRA accounted for approximately two-thirds of all disability worsening events, in line with recent evidence in relapsing MS patients from randomized controlled trials [3, 4] and real-world cohorts [4–8], indicating PIRA as the main driver of disability accumulation in MS. Moreover, in our study, PIRA was more frequently associated with disability accumulation that persisted at the end of the follow-up. This implies a greater impact of PIRA on the one hand, as it has been recently described by Tur and colleagues [8], who demonstrated that having PIRA after a first demyelinating event was related with an unfavorable long-term prognosis, especially if it occurs early in the disease course. On the other hand, this finding could be, at least partly, explained by higher recovery potential in case of RAW. Indeed, in a recent analysis of the CombiRx dataset [14], 84% of patients with relapses experienced disability recovery, mostly within 180 days from relapse onset. This proportion reduced to 52–55% when relapse recovery was confirmed at 12 and 24 weeks, figure very close to that observed in our cohort.

As for the transition to SPMS, it occurred in 27.6% of patients, mostly at their first PIRA. The risk of transition was higher after a sustained PIRA event. While it is well acknowledged that PIRA is associated with SPMS since, per definition, progressive phase onsets with a PIRA (all SPMS transitions are PIRA events), our data hold several implications. A single PIRA event is sufficient to starts progression in most of the patients (73.4%), particularly in case of sustained PIRA. This is in line with the greater impact of PIRA, as indicated by the high proportion of SDA and confirms its detrimental prognostic role. It has to be noted the less than one-third of patient with sustained PIRA did not fulfill our definition of SPMS at the end of the follow-up. These patients were more frequently female, less disabled, with shorter disease duration, shorter follow-up duration. It is possible to speculate that shorter follow-up duration, and lower EDSS prevented the achievement of the definition of SP that requires a EDSS score of at least 4.0. We therefore believe that the occurrence of any sustained PIRA could represent the onset of the progressive phase. Notably, however, in this subgroup of patients, the percentage of time spent on DMT was higher, indicating a potential effectiveness of treatment in reducing the risk of progression. On the other hand, not all PIRA events are transitions to SPMS. This is particularly relevant in case of non-sustained PIRA. Nevertheless, 25% of patients with non-sustained PIRA can be classified as SPMS at the end of the follow-up, especially in older subjects, with higher disability levels, and longer disease duration. Therefore, any PIRA (sustained or not) occurring in patients with these characteristics could herald the transition to SPMS.

Overall, when validated against a robust outcome such as transition to an algorithm-based diagnosis of SPMS, sustained PIRA appears to be the best definition of disability accumulation independent of relapse activity, at least in terms of specificity. This finding is relevant to the ongoing debate on the appropriate definition of PIRA [15], showing that persistence of disability accrual at the end of the follow-up is the best time interval for confirmation of progression. Unfortunately, while sustained PIRA probably represents a more accurate identification, it is of limited application in clinical practice. Sustained PIRA is an a posteriori definition, requiring a long period of observation (years) for its confirmation, and appears unsuitable to guide therapeutic decisions.

Moreover, since PIRA is frequent even in the earliest phases of the disease, the onset of progression is expected to occur earlier than previously estimated, confirming the well acknowledged delay in the identification of SP [16]. Furthermore, the occurrence of PIRA is quite invariably associated with irreversible disability, highlighting the need of prevention. In this regard, elucidating pathogenetic underpinnings and identifying reliable and early risk factors of PIRA are warranted.

Importantly, in all the analyses higher exposure to DMT reduce the risk of any CDA, both RAW and PIRA, as well as the risk of transition to SPMS. This finding replicates in real-world data that emerged from the pooled analysis of OPERA-1 and OPERA-2 trials [3] and suggests that pathogenetic mechanisms sustaining PIRA can be, at least in part, modified by currently approved DMTs for relapsing MS.

Focusing on the subpopulation with at least 2 confirmed disability accrual events during follow-up, the great majority of patients had at least one PIRA event (only PIRA in 49.8% of patients and RAW + PIRA events in 43.6%). In the subgroup of subjects with mixed type of CDA events, RAW and PIRA were variably interwoven during follow-up, with RAW becoming less likely to occur over time. Indeed, across the follow-up of these patients, PIRA events were progressively more represented from their first through the last CDA event. The occurrence of PIRA was related with older age, and higher disability levels at baseline. Only 6.6% of patients experienced exclusively RAW events; these patients were younger, more frequently female, with lower levels of disability at baseline, and with higher rates of relapses during follow-up, defining a subgroup of patients with a more “inflammatory” phenotype.

In addition, a key determinant in the way by which CDA occurs appears to be the age, with RAW events being more frequent in younger patients and PIRA events in older patients, in line with past observations [7, 8, 17]. However, a recent assessment of RAW and PIRA in a pediatric-onset MS (POMS) population showed that, although rarely detectable before 18 years of age, PIRA occurred even in young POMS patients, indicating that other factors beyond age are involved in PIRA appearance [18].

The interpretation of the study findings should take into account a few limitations. CDA events were identified using the study entry EDSS as reference baseline, while the use of a roving baseline demonstrated higher sensitivity and accuracy. [6] Moreover, the analysis of factors associated with disability worsening was limited to the EDSS score alone. In a previous observation [3], PIRA was largely driven by other disability measures, such as the Timed 25-Foot Walk Test and the 9-Hole Peg Test. Therefore, an underestimation of PIRA events in our sample cannot be excluded. On the other hand, we cannot exclude that unnoticed (milder) relapses or MRI inflammatory activity may have contributed to PIRA events, especially the transient ones. As our analysis did not include MRI data, it is possible that we might have missed some PIRA event with a subclinical “relapsing” activity at brain and/or spinal MRI. However, in a previous assessment taking into account MRI examinations, true PIRA (progression independent of relapse and MRI activity) remained the main determinant of disability accumulation in relapsing MS [7].

Despite these limitations, our data add and expand previous observations on silent progression in MS and are consistent with the view of the disease as a single continuum, in which RAW and PIRA co-occur since the earliest phases, with age representing the main determinant of disease phenomenology. In particular, focusing on multiple CDA events, PIRA emerges as the main driver of disability accumulation in relapsing-onset MS and is associated with a worse prognosis and higher levels of disability in the long term. In addition, our study extends our knowledge on the relationship between PIRA and SPMS. The great majority of sustained PIRA, as well as non-sustained PIRA in older patients with longer disease duration and greater disability are linked to transition to SP and could therefore represent a criterion to mark, on clinical grounds, the putative onset of the progressive phase. Moreover, as for the identification of the most specific definition of PIRA, sustained disability appears to be the more accurate time interval for its confirmation, although it is of limited application in clinical practice. On the other hand, earlier and longer exposure to DMTs reduces the risk of any CDA both RAW and PIRA. Therefore, deepening our knowledge on PIRA pathogenesis and risk factors remains crucial in order to refine therapeutic interventions for MS subjects at higher risk of disability accumulation.

## Supplementary Information

Below is the link to the electronic supplementary material.

Supplementary file1 (DOCX 46 KB)

## Data Availability

Anonymized data, not published in the article, will be shared on reasonable request from a qualified investigator.
