# Correction to: Progression independent of relapse activity in relapsing multiple sclerosis: impact and relationship with secondary progression

**DOI:** 10.1007/s00415-024-12627-3

**Published:** 2024-09-04

**Authors:** Emilio Portaccio, Matteo Betti, Ermelinda De Meo, Ilaria Addazio, Luisa Pastò, Lorenzo Razzolini, Rocco Totaro, Daniele Spitaleri, Alessandra Lugaresi, Eleonora Cocco, Marco Onofrj, Franco Di Palma, Francesco Patti, Davide Maimone, Paola Valentino, Valentina Torri Clerici, Alessandra Protti, Diana Ferraro, Giacomo Lus, Giorgia Teresa Maniscalco, Vincenzo Brescia Morra, Giuseppe Salemi, Franco Granella, Ilaria Pesci, Roberto Bergamaschi, Umberto Aguglia, Marika Vianello, Marta Simone, Vito Lepore, Pietro Iaffaldano, Giancarlo Comi, Massimo Filippi, Maria Trojano, Maria Pia Amato, the Italian Multiple Sclerosis Register

**Affiliations:** 1 Department of NEUROFARBA, University of Florence, Careggi University Hospital, Florence, Italy; 2 NMR Research Unit, Queen Square Multiple Sclerosis Centre, UCL Queen Square Institute of Neurology, University College London, London, UK; 3 San Salvatore Hospital, L’Aquila, Italy; 4 AORN San G. Moscati, Avellino, Italy; 5 IRCCS Istituto Delle Scienze Neurologiche Di Bologna, Bologna, Italy; 6 Dipartimento Di Scienze Biomediche E Neuromotorie, Università Di Bologna, Bologna, Italy; 7 University of Cagliari, Cagliari, Italy; 8 University G. d’Annunzio Di Chieti-Pescara, Chieti, Italy; 9 ASST Lariana Ospedale S. Anna, Como, Italy; 10 University of Catania, Catania, Italy; 11 UOS Sclerosi Multipla, Policlinico G Rodolico-San Marco, University of Catania, Catania, Italy; 12 Centro Sclerosi Multipla, Azienda Ospedaliera Cannizzaro, Catania, Italy; 13 Institute of Neurology, University Magna Graecia, Catanzaro, Italy; 14 Fondazione IRCCS Istituto Neurologico C. Besta, Milan, Italy; 15 Niguarda Hospital, Milan, Italy; 16 Department of Neurosciences, Ospedale Civile Di Baggiovara, Azienda Ospedaliero-Universitaria Di Modena, Modena, Italy; 17 University of Campania Luigi Vanvitelli, Naples, Italy; 18 A Cardarelli Hospital, Naples, Italy; 19 Federico II University, Naples, Italy; 20 Department of Biomedicine, Neuroscience and Advanced Diagnostics (BIND), University of Palermo, Palermo, Italy; 21 University of Parma, Parma, Italy; 22 Ospedale VAIO Di Fidenza AUSL PR, Fidenza (PR), Italy; 23 IRCCS Fondazione Mondino, Pavia, Italy; 24 Department of Medical and Surgical Sciences, Magna Graecia University of Catanzaro, Catanzaro, Italy; 25 Ca’ Fancello Hospital, AULSS2, Treviso, Italy; 26 Pediatric MS Center, Department of Precision and Regenerative Medicine and Ionian Area, University of Bari Aldo Moro, Bari, Italy; 27 Istituto Di Ricerche Farmacologiche Mario Negri IRCCS, Milan, Italy; 28 Department of Translational Biomedicine and Neurosciences, University of Bari Aldo Moro, DiBraiN, Bari, Italy; 29 Casa Di Cura del Policlinico, Vita-Salute San Raffaele University, Milan, Italy; 30 Vita-Salute San Raffaele University, Milan, Italy; 31 IRCCS San Raffaele Scientific Institute, Milan, Italy; 32 IRCCS Don Carlo Gnocchi Foundation, Florence, Italy

**Correction to: Journal of Neurology** 10.1007/s00415-024-12448-4

In the original version of this article, affiliation details for author Maria Pia Amato were incorrectly linked to all affiliations but should have been

Department of NEUROFARBA, University of Florence, Careggi University Hospital, Florence, Italy

IRCCS Don Carlo Gnocchi Foundation, Florence, Italy

And from affiliation number 26 to the last affiliation, the affiliation number order is not correct. The correct authors and affiliations should be

**Authors and Affiliations**

Emilio Portaccio^1^ · Matteo Betti^1^ · Ermelinda De Meo^1,2^ · Ilaria Addazio^1^ · Luisa Pastò^1^ · Lorenzo Razzolini^1^ · Rocco Totaro^3^ · Daniele Spitaleri^4^ · Alessandra Lugaresi^5,6^ · Eleonora Cocco^7^ · Marco Onofrj^8^ · Franco Di Palma^9^ ·Francesco Patti^10,11^ · Davide Maimone^12^ · Paola Valentino^13^ · Valentina Torri Clerici^14^ · Alessandra Protti^15^ ·Diana Ferraro^16^ · Giacomo Lus^17^ · Giorgia Teresa Maniscalco^18^ · Vincenzo Brescia Morra^19^ · Giuseppe Salemi^20^ · Franco Granella^21^ · Ilaria Pesci^22^ · Roberto Bergamaschi^23^ · Umberto Aguglia^24^ · Marika Vianello^25^ · Marta Simone^26^ · Vito Lepore^27^ · Pietro Iaffaldano^28^ · Giancarlo Comi^29^ · Massimo Filippi^30,31^ · Maria Trojano^28^ · Maria Pia Amato^1,32^ on behalf of the Italian Multiple Sclerosis Register

^1^Department of NEUROFARBA, University of Florence, Careggi University Hospital, Florence, Italy

^2^NMR Research Unit, Queen Square Multiple Sclerosis Centre, UCL Queen Square Institute of Neurology, University College London, London, UK

^3^San Salvatore Hospital, L’Aquila, Italy

^4^AORN San G. Moscati, Avellino, Italy

^5^IRCCS Istituto Delle Scienze Neurologiche Di Bologna, Bologna, Italy

^6^Dipartimento Di Scienze Biomediche E Neuromotorie, Università Di Bologna, Bologna, Italy

^7^University of Cagliari, Cagliari, Italy

^8^University G. d’Annunzio Di Chieti-Pescara, Chieti, Italy

^9^ASST Lariana Ospedale S. Anna, Como, Italy

^10^University of Catania, Catania, Italy

^11^UOS Sclerosi Multipla, Policlinico G Rodolico-San Marco, University of Catania, Catania, Italy

^12^Centro Sclerosi Multipla, Azienda Ospedaliera Cannizzaro, Catania, Italy

^13^Institute of Neurology, University Magna Graecia, Catanzaro, Italy

^14^Fondazione IRCCS Istituto Neurologico C. Besta, Milan, Italy

^15^Niguarda Hospital, Milan, Italy

^16^Department of Neurosciences, Ospedale Civile Di Baggiovara, Azienda Ospedaliero-Universitaria Di Modena, Modena, Italy

^17^University of Campania Luigi Vanvitelli, Naples, Italy

^18^A Cardarelli Hospital, Naples, Italy

^19^Federico II University, Naples, Italy

^20^Department of Biomedicine, Neuroscience and Advanced Diagnostics (BIND), University of Palermo, Palermo, Italy

^21^University of Parma, Parma, Italy

^22^Ospedale VAIO Di Fidenza AUSL PR, Fidenza (PR), Italy

^23^IRCCS Fondazione Mondino, Pavia, Italy

^24^Department of Medical and Surgical Sciences, Magna Graecia University of Catanzaro, Catanzaro, Italy

^25^Ca’ Fancello Hospital, AULSS2 Treviso, Italy

^26^Pediatric MS Center, Department of Precision and Regenerative Medicine and Ionian Area, University of Bari Aldo Moro, Bari, Italy

^27^Istituto Di Ricerche Farmacologiche Mario Negri IRCCS, Milan, Italy

^28^Department of Translational Biomedicine and Neurosciences, University of Bari Aldo Moro, DiBraiN, Bari, Italy

^29^Casa Di Cura del Policlinico, Vita-Salute San Raffaele University, Milan, Italy

^30^Vita-Salute San Raffaele University, Milan, Italy

^31^IRCCS San Raffaele Scientific Institute, Milan, Italy

^32^IRCCS Don Carlo Gnocchi Foundation, Florence, Italy

