# Supplementary material for: Progression independent of relapse activity in relapsing multiple sclerosis: impact and relationship with secondary progression
Source: J Neurol. 2024 May 28;271(8):5074–82. doi: 10.1007/s00415-024-12448-4 (PMC11319422; doi:10.1007/s00415-024-12448-4)
Supplement: Supplementary file 1 — Supplementary file1 (DOCX 46 KB) [file 415_2024_12448_MOESM1_ESM.docx]

**Journal of Neurology**

**Progression independent of relapse activity in relapsing multiple sclerosis: impact and relationship with secondary progression**

Emilio Portaccio^1*^, Matteo Betti^1*^, Ermelinda De Meo^1,2^, Ilaria Addazio^1^, Luisa Pastò^1^, Lorenzo Razzolini^1^, Rocco Totaro*^3^*, Daniele Spitaleri*^4^*, Alessandra Lugaresi*^5,6^*, Eleonora Cocco*^7^*, Marco Onofrj*^8^*, Franco Di Palma*^9^*, Francesco Patti*^10,11^*, Davide Maimone*^12^*, Paola Valentino*^13^*, Valentina Torri Clerici*^14^*, Alessandra Protti*^15^*, Diana Ferraro*^16^*, Giacomo Lus*^17^* , Giorgia Teresa Maniscalco*^18^* , Vincenzo Brescia Morra*^19^* , Giuseppe Salemi*^20^* , Franco Granella*^21^* , Ilaria Pesci*^22^* , Roberto Bergamaschi*^23^* , Umberto Aguglia*^24^* , Marika Vianello*^25^* , Marta Simone*^26^* , Vito Lepore*^27^* , Pietro Iaffaldano*^26^* , Giancarlo Comi*^28^*, Massimo Filippi*^29,30^* , Maria Trojano*^26^*, and Maria Pia Amato*^1-31^* , for the Italian Multiple Sclerosis Register.

* Contributed equally, two first authors

¹University of Florence, Florence, Italy, Careggi University Hospital, Florence, Italy,

^2^NMR Research Unit, Queen Square Multiple Sclerosis Centre, UCL Queen Square Institute of Neurology, University College London, London UK

^3^San Salvatore Hospital, L'Aquila, Italy,

^4^AORN San G. Moscati, Avellino, Italy,

^5^IRCCS Istituto delle Scienze Neurologiche di Bologna, Bologna, Italy,

^6^Dipartimento di Scienze Biomediche e Neuromotorie - Università di Bologna, Bologna, Italy,

^7^University of Cagliari, Cagliari, Italy,

^8^University G. d’Annunzio di Chieti-Pescara, Chieti, Italy,

^9^ASST Lariana Ospedale S. Anna, Como, Italy,

^10^University of Catania, Catania, Italy,

^11^UOS Sclerosi Multipla, Policlinico G Rodolico-San Marco, University of Catania

^12^Centro Sclerosi Multipla, Azienda Ospedaliera Cannizzaro, Catania, Italy,

^13^Institute of Neurology, University "Magna Graecia", Catanzaro, Italy,

^14^Fondazione IRCCS Istituto Neurologico C. Besta, Milan, Italy,

^15^Niguarda Hospital, Milan, Italy,

^16^Department of Neurosciences, Ospedale Civile di Baggiovara, Azienda Ospedaliero-Universitaria di Modena, Modena, Italy,

^17^University of Campania Luigi Vanvitelli, Naples, Italy,

^18^A Cardarelli Hospital, Naples, Italy,

^19^Federico II University, Naples, Italy,

^20^Department of BIomedicine, Neuroscience and advanced Diagnostics (BIND), University of Palermo, Palermo, Italy

^21^University of Parma, Parma, Italy,

^22^Ospedale VAIO di Fidenza AUSL PR, Fidenza (PR), Italy,

^23^IRCCS Fondazione Mondino, Pavia, Italy,

^24^Department of Medical and Surgical Sciences, Magna Graecia University of Catanzaro, Italy

^25^Ca' Fancello Hospital, AULSS2, Treviso, Italy,

^26^University of Bari Aldo Moro , Department of Translational Biomedicine and Neurosciences – DiBraiN, Bari, Italy

^27^Istituto di Ricerche Farmacologiche Mario Negri IRCCS, Milan, Italy,

^28^Casa di Cura del Policlinico, Vita-Salute San Raffale University, Milan, Italy,

^29^Vita-Salute San Raffaele University, Milan, Italy,

^30^IRCCS San Raffaele Scientific Institute, Milan, Italy,

^31^IRCCS Don Carlo Gnocchi Foundation, Florence, Italy.

**Corresponding author:**

Emilio Portaccio, MD

Department of NEUROFARBA

University of Florence, Florence, Italy

e-mail: emilio.portaccio@unifi.it

ORCID: 0000-0002-9662-1762**Supplementary Figure S1 Percentage of RAW and PIRA in the whole sample and by number of CDA events**

**Legend to Supplementary Figure S1.**

RAW: Relapse Associated Worsening; PIRA: Progression Independent of Relapse Activity; CDA: Confirmed Disability Accrual

**Supplementary Table S1. Characteristics of patients with sustained PIRA event (n=5476)**

|  | **Total (n=5476)** | **SP (n=4010)** | **Non-SP (n=1466)** | **P** |
| --- | --- | --- | --- | --- |
| **Age at baseline, year, mean + SD** | 38.7+10.7 | 39.3+10.8 | 36.9+10.2 | <0.001 |
| **Age at onset, year, mean + SD** | 31.5+10.3 | 31.6+10.4 | 31.2+10.0 | 0.134 |
| **Sex, female n (%)** | 3589 (65.6) | 2582 (64.4) | 1007 (68.7) | 0.003 |
| **Disease course , n (%)**  **CIS**  **RR** | 756 (13.8)  4720 (86.2) | 512 (12.8)  3498 (87.2) | 244 (16.6)  1222 (83.4) | <0.001 |
| **Disease duration, year, median (IQR)** | 4.4 (1.2-10.7) | 5.1 (1.6-11.4) | 2.8 (0.7-8.7) | <0.001 |
| **EDSS, median (IQR)** | 2.0 (1.0-3.5) | 2.5 (2.0-3.5) | 1.0 (0-1.5) | <0.001 |
| **Onset topography, n (%)**  **Unifocal**  **Multifocal** | 4702 (85.9)  774 (14.1) | 3413 (85.1)  597 (14.9) | 1289 (87.9)  177 (12.1) | 0.008 |
| **Follow-up duration, year, mean + SD** | 13.2+5.9 | 13.8+6.1 | 11.7+5.1 | <0.001 |
| **Number of visits before SP, mean + SD** | 15.7+13.3 | 13.9+12.4 | 20.4+14.5 | <0.001 |
| **Number of relapses before SP, median (IQR)** | 4 (2-6) | 4 (2-7) | 3 (2-5) | <0.001 |
| **Percentage of follow-up spent on DMT before SP, mean + SD** | 64.9+38.1 | 63.1+38.9 | 69.6+35.4 | <0.001 |
| **Final EDSS, median (IQR)** | 6.0 (4.0-6.5) | 6.5 (5.5-7.0) | 3.0 (2.0-3.5) | <0.001 |

**Legend to Supplementary Table S1.**

PIRA: Progression Independent of Relapse Activity; SP: Secondary Progression; SD: Standard Deviation; CIS: Clinically Isolated Syndrome; RR: Relapsing-Remitting; IQR: Inter-Quartile Range; EDSS: Expanded Disability Status Scale; DMT: Disease Modifying Treatment

**Supplementary Table S2. Characteristics of patients with non-sustained PIRA event (n=1790)**

|  | **Total (n=1790)** | **SP (n=443)** | **Non-SP (n=1347)** | **P** |
| --- | --- | --- | --- | --- |
| **Age at baseline, year, mean + SD** | 35.8+10.2 | 38.5+10.1 | 34.9+10.0 | <0.001 |
| **Age at onset, year, mean + SD** | 29.5+9.4 | 30.0+9.5 | 29.4+9.4 | 0.212 |
| **Sex, female n (%)** | 1282 (71.6%) | 315 (71.1) | 967 (71.8) | 0.782 |
| **Disease course , n (%)**  **CIS**  **RR** | 245 (13.7)  1545 (86.3) | 56 (12.6)  387 (87.4) | 189 (14.0)  1158 (86.0) | 0.460 |
| **Disease duration, year, median (IQR)** | 3.3 (1.0-9.6) | 5.8 (2.0-12.5) | 2.8 (0.8-8.1) | <0.001 |
| **EDSS, median (IQR)** | 1.5 (1.0-2.5) | 3.0 (2.0-4.0) | 1.5 (1.0-2.0) | <0.001 |
| **Onset topography, n (%)**  **Unifocal**  **Multifocal** | 1563 (87.3)  227 (12.7) | 383 (86.5)  60 (13.5) | 1180 (87.6)  167 (12.4) | 0.529 |
| **Follow-up duration, year, mean + SD** | 13.4+5.5 | 14.8+6.4 | 12.9+5.0 | <0.001 |
| **Number of visits before SP, mean + SD** | 26.3+17.5 | 16.9+13.9 | 29.3+17.5 | <0.001 |
| **Number of relapses before SP, median (IQR)** | 4 (2-7) | 5 (3-9) | 4 (2-7) | <0.001 |
| **Percentage of follow-up spent on DMT before SP, mean + SD** | 75.0+32.1 | 68.2+36.3 | 77.2+30.2 | <0.001 |
| **Final EDSS, median (IQR)** | 2.5 (1.5-4.0) | 5.5 (4.5-6.5) | 2.0 (1.5-3.0) | <0.001 |

**Legend to Supplementary Table S2.**

PIRA: Progression Independent of Relapse Activity; SP: Secondary Progression; SD: Standard Deviation; CIS: Clinically Isolated Syndrome; RR: Relapsing-Remitting; IQR: Inter-Quartile Range; EDSS: Expanded Disability Status Scale; DMT: Disease Modifying Treatment

**Supplementary Table S3. Factors associated with only RAW events in patients with at least 2 CDA (n=4217)**

|  | **OR** | **95% CI** | **p** |
| --- | --- | --- | --- |
| **Sex (female versus male)** | 1.47 | 1.10-1.97 | 0.011 |
| **Onset topography (multifocal versus unifocal)** | 0.89 | 0.61-1.30 | 0.559 |
| **Age** | 0.97 | 0.96-0.99 | 0.001 |
| **Disease course (RR vs CIS)** | 0.81 | 0.56-1.18 | 0.226 |
| **Disease duration** | 1.01 | 0.99-1.03 | 0.420 |
| **EDSS** | 0.87 | 0.79-0.97 | 0.009 |
| **Percentage of time spent on DMT during follow-up** | 1.18 | 0.74-1.87 | 0.491 |
| **Number of relapses during follow-up** | 1.21 | 1.17-1.24 | <0.001 |
| **Number of EDSS evaluations during follow-up** | 0.99 | 0.98-1.01 | 0.333 |
| **Follow-up duration** | 0.89 | 0.86-0.92 | <0.001 |

**Legend to Supplementary Table S3.**

RAW: Relapse Associated Worsening; CDA: Confirmed Disability Accrual; RR: Relapsing-Remitting; CIS: Clinically Isolated Syndrome; EDSS: Expanded Disability Status Scale; DMT: Disease Modifying Treatment

**Supplementary Table S4. Factors associated with only PIRA events in patients with at least 2 CDA (n=4217)**

|  | **OR** | **95% CI** | **p** |
| --- | --- | --- | --- |
| **Sex (female versus male)** | 1.08 | 0.93-1.26 | 0.304 |
| **Onset topography (multifocal versus unifocal)** | 1.11 | 0.91-1.36 | 0.315 |
| **Age** | 1.01 | 1.01-1.02 | 0.036 |
| **Disease course (RR vs CIS)** | 0.95 | 0.76-1.18 | 0.649 |
| **Disease duration** | 1.00 | 0.99-1.02 | 0.512 |
| **EDSS** | 1.15 | 1.10-1.21 | <0.001 |
| **Percentage of time spent on DMT during follow-up** | 1.41 | 1.12-1.78 | 0.004 |
| **Number of relapses during follow-up** | 0.69 | 0.67-0.71 | <0.001 |
| **Number of EDSS evaluations during follow-up** | 1.00 | 0.99-1.01 | 0.672 |
| **Follow-up duration** | 1.05 | 1.04-1.07 | <0.001 |

**Legend to Supplementary Table S4.**

PIRA: Progression Independent of Relapse Activity; CDA: Confirmed Disability Accrual; RR: Relapsing-Remitting; CIS: Clinically Isolated Syndrome; EDSS: Expanded Disability Status Scale; DMT: Disease Modifying Treatment
